# Regulation of 53BP1 Protein Stability by RNF8 and RNF168 Is Important for Efficient DNA Double-Strand Break Repair

**DOI:** 10.1371/journal.pone.0110522

**Published:** 2014-10-22

**Authors:** Yiheng Hu, Chao Wang, Kun Huang, Fen Xia, Jeffrey D. Parvin, Neelima Mondal

**Affiliations:** 1 Department of Biomedical Informatics, The Ohio State University, Columbus, Ohio, United States of America; 2 Department of Radiation Oncology, The Ohio State University, Columbus, Ohio, United States of America; 3 School of Life Sciences, Jawaharlal Nehru University, New Delhi, India; The University of Hong Kong, Hong Kong

## Abstract

53BP1 regulates DNA double-strand break (DSB) repair. In functional assays for specific DSB repair pathways, we found that 53BP1 was important in the conservative non-homologous end-joining (C-NHEJ) pathway, and this activity was dependent upon RNF8 and RNF168. We observed that 53BP1 protein was diffusely abundant in nuclei, and upon ionizing radiation, 53BP1 was everywhere degraded except at DNA damage sites. Depletion of RNF8 or RNF168 blocked the degradation of the diffusely localized nuclear 53BP1, and ionizing radiation induced foci (IRIF) did not form. Furthermore, when 53BP1 degradation was inhibited, a subset of 53BP1 was bound to DNA damage sites but bulk, unbound 53BP1 remained in the nucleoplasm, and localization of its downstream effector RIF1 at DSBs was abolished. Our data suggest a novel mechanism for responding to DSB that upon ionizing radiation, 53BP1 was divided into two populations, ensuring functional DSB repair: damage site-bound 53BP1 whose binding signal is known to be generated by RNF8 and RNF168; and unbound bulk 53BP1 whose ensuing degradation is regulated by RNF8 and RNF168.

## Introduction

DNA double-strand break (DSB) repair involves two major pathways: homologous recombination (HR) and nonhomologous end-joining (NHEJ). HR has a major homology-directed repair (HDR) pathway, which is a relatively precise form of repair and a minor subpathway called single-strand annealing (SSA), which causes DNA resection until homology at repair junctions is revealed [1]. To date, two types of end-joining systems are defined in the NHEJ: the major one is the conservative-NHEJ (C-NHEJ), which is predominantly associated with precise joining of DSB ends without altering the DNA sequence [2]. The alternative pathway for NHEJ (Alt-NHEJ) is highly mutagenic since it catalyzes DNA resection and utilizes imperfect microhomology for end-joining partners and thus resulting in deletions at repair junctions [3].

53BP1 is known to promote the repair of DSBs by NHEJ [4]–[9]. 53BP1 deletion in mouse results in a severe defect in class-switch recombination, a process dependent on NHEJ and associated with increased DNA end resection at the IgH locus [10]–[12]. Loss of 53BP1 restores homologous recombination in BRCA1-deficient murine cells, indicating that 53BP1 inhibits DNA resection in DSB repair, by the regulation of the downstream effector RIF1 to control 5′ end resection [13]–[15]. Since it is known that 53BP1 directly regulates efficient total NHEJ repair events in mammalian cells [16] and 53BP1 inhibits end resection, it is predictable that 53BP1 might favor the C-NHEJ pathway over Alt-NHEJ.

Upon DNA DSB induction, a cascade of protein modification and relocalization is triggered: phosphorylation of H2AX (γ-H2AX) results in the recruitment of downstream factors, such as the E3 ubiquitin ligases RNF8 and RNF168, leading to the formation of K63-linked polyubiquitin chains on histones at DSBs. This ubiquitination cascade regulated by RNF8 and RNF168 is responsible for the localization of repair mediators, including BRCA1 and 53BP1 to the DNA damage sites [17]–[27]. Localization of 53BP1 to DSBs involves its recognition of H2A ubiquitinated on Lys-15 (H2AK15ub), the latter being a product of RNF168 via its ubiquitination-dependent recruitment (UDR) motif binding to K63-linked ubiquitination on chromatin. 53BP1 binding to the chromatin at the damage sites also requires dimethylation of histone H4 on lysine 20 (H4K20me2) via the 53BP1 tandem Tudor domain [28]–[31] plus a K63-linked ubiquitination of the 53BP1 protein at lysine 1268 by RNF168 [32].

In this study, we identified that 53BP1 acts specifically to promote conservative-NHEJ in a RNF8- and RNF168-dependent manner. We found that RNF8 and RNF168 not only mark histones at the break site to create a 53BP1 binding site, but these ubiquitin ligases also regulate the proteasome-mediated degradation of 53BP1. Failure to degrade 53BP1 protein not bound to DSBs leads to mislocalization of a downstream factor RIF1, thus impairing DSB repair.

## Materials and Methods

### Antibodies and reagents

We used the following primary antibodies: anti-53BP1 (Santa Cruz, H-300, for immunoblot and immunofluorescence), anti-RAD51 (Santa Cruz, H-92), anti-γ-H2AX (Millipore, clone JBW301, for immunoblot and Immunofluorescence), anti-RIF1 (Santa Cruz, N-20, for immunoblot and Immunofluorescence), anti-RNF8 (Abnova), anti-RNF168 (Abcam), anti-α-tubulin (Sigma), anti-H4 (Millipore), anti-β-actin (Cell Signaling), anti-HA (purified from mouse ascites fluid), and anti-RHA (purified from rabbit serum). MG132 (Enzo life Sciences, dissolved in DMSO, treated 30 min prior to irradiation), caffeine (Sigma, dissolved in ddH_2_O, treated 1 h prior to irradiation), cycloheximide (Fluka Analytical, dissolved in ethanol, treated 15 min prior to irradiation).

### HR and end-joining assays

HDR, SSA and Alt-NHEJ were performed as previously described [33]–[36]. The HeLa-derived cell lines that stably integrate the HDR and SSA recombination substrates have been described [35]. The Alt-NHEJ substrate [33] was stably integrated into the HeLa genome. For the DSB repair assays, the appropriate cell line was transfected with siRNAs and, if necessary for the experiment, an expression plasmid on day 1. On day 3 the cells were re-transfected plus a plasmid expressing the I-SceI endonuclease, which initiates a DSB lesion. The amount of repair activity was determined by counting the percentage of GFP-positive cells using flow cytometry.

The C-NHEJ assay utilized quantitative real-time PCR and was carried out as described in [37] with the following modification. The genomic DNA isolated 3 days after transfection of the I-SceI plasmid was treated with the restriction enzyme XhoI and purified by Qiagen PCR purification kit before real-time PCR was applied. RPS17 probe (Hs00734303_g1, Applied Biosystems) was used as an internal control and quantitative ΔΔC_T_ method was used to analyze the data.

### RNA interference and plasmids

We used the following siRNAs produced by Sigma: siControl targeting the luciferase gene: 5′-CGUACGCGGAAUACUUCGA-3′ [34]; si53BP1: 5′-GAAGGACGGAGUACUAAUA-3′ [38]; si53BP1-2 starting at nucleotide 6051: 5′-UACUUGGUCUUACUGGUUU-3′; siRNF8: 5′-GGACAAUUAUGGACAACAA-3′ [39]; siRNF168: 5′-GGCGAAGAGCGAUGGAGGA-3′ [38]; siLigase IV: 5′-AGGAAGUAUUCUCAGGAAUUA-3′ [38]; siBRCA1: 5′-GCUCCUCUCACUCUUCAGU-3′ [34]; siBRCA2: 5′-UAAAUUUGGACAUAAGGAGUCCUCC-3′ [34]. HA-tagged wild-type 53BP1 expression plasmid was a kind gift from Kuniyoshi Iwabuchi (Kanazawa Medical University). I-SceI expression plasmid has been previously described [34] and was a kind gift from Maria Jasin (Memorial Sloan-Kettering Cancer Institute). The total siRNA amount was adjusted to be the same in each sample by adding siControl. All RNAi transfections were carried out using Oligofectamine (Life Technologies) and plasmid transfections were using Lipofectamine2000 (Life Technologies).

### Preparation of whole cell lysates

Whole cell extracts (if not otherwise indicated) were prepared by lysing cells in cell extraction buffer (50 mM Tris, pH 7.9, 300 mM NaCl, 0.5% Nonidet-40, 1 mM EDTA, 5% glycerol, 1 mM phenylmethylsulfonyl fluoride, 1 mM dithiothreitol, 1X complete protease inhibitor cocktail from Sigma). Alternatively, when indicated, the whole cell lysates were either prepared by direct boiling in sodium dodecyl sulfate (SDS) containing buffer (2% SDS in phosphate-buffered saline; PBS) and sonicated five times using a Fisher Scientific probe sonicator for 10 s pulses each at 45% amplitude; or cells were lysed and boiled in urea containing buffer (8 M urea and 2% SDS in PBS), and sonicated as above.

### Immunofluorescence microscopy

Cells were fixed with cold 4% paraformaldehyde for 15 min and permeabilized with cold 70% ethanol for 5 min before blocking in 8% bovine serum albumin/PBS for 1 h. Primary antibodies were diluted at 1∶500 (the rabbit γ-H2AX antibody was used at 1∶1000 dilution) for incubation at room temperature for 2 h. Cells were washed with PBS and stained with secondary antibodies. DAPI was then added at 1: 10,000 for 5 min to stain the nucleus. For experiments in which the cells were extracted with NP40 prior to fixation, the extraction buffer (same as the cell extraction buffer above) was applied to cells that had been washed in PBS, followed by the above protocol for fixation and staining. Images were viewed and acquired using the 60X oil objective lens with a Zeiss Axiovert 200 M microscope. Fluorescence signals for the same indicated protein were captured on the same day with the same exposing times for all samples using software AxioVision 4.8.

### Image analysis

For the immunofluorescence images, relative 53BP1 signal within the nuclei in each sample was assessed using ImageJ software (NIH). Briefly, 30 or 100 nuclei were assessed, depending on the different cell line. Mean intensity gray values of 53BP1 signal at foci regions in each nucleus in irradiated samples were scored and normalized according to the mean intensity of diffuse 53BP1 signal in unirradiated sample.

Alternatively, quantitative image analysis of 53BP1 protein signal in the nuclei was carried out using an algorithm in MATLAB R2013a. The program code is available on request. Briefly, 80–100 cells in each sample were randomly selected. DAPI stain was used to segment the outlines of nuclei and masked on the 53BP1 stain to measure the 53BP1 signal within the nuclei mask area. The distribution of all individual pixel intensity values of 53BP1 signal (*X* axis) in the 80–100 nuclei was then plotted against the total number of pixels (*Y* axis) in each sample and compared among the samples. Distribution of pixel intensity in each cohort in the 80–100 nuclei was examined using histogram with the x-axis being for intensities of 0 to 4096, (binsize  = 1).

### Statistical analysis

Data were objectively compared between different groups for each sample using unpaired and two-tailed Student's t test (*, **, and *** represent p<0.05, p<0.01, and p<0.001, respectively).

## Results

### 53BP1 functions in conservative-NHEJ dependent on RNF8 and RNF168

**Figure 1:**
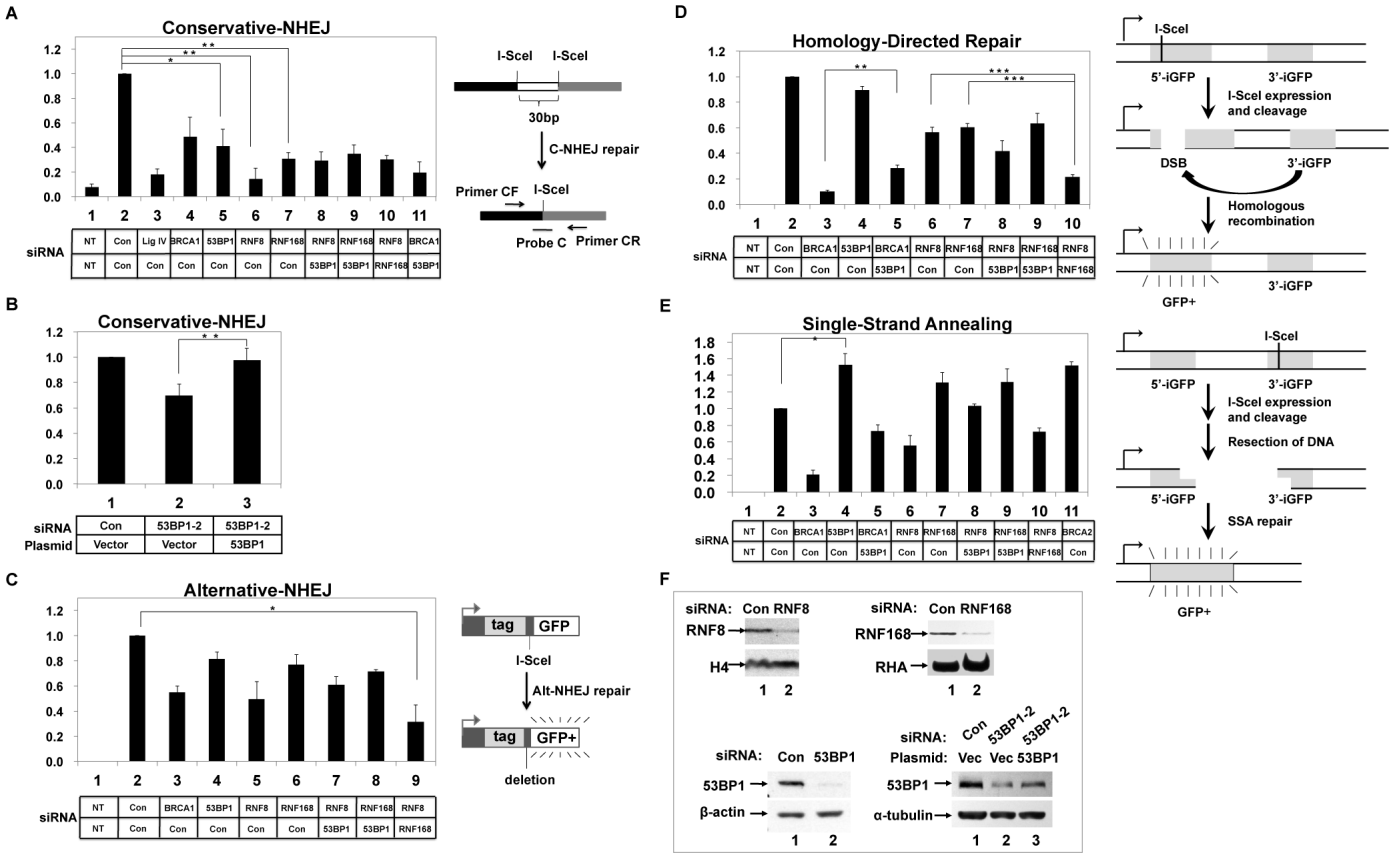
53BP1 function in conservative-NHEJ is dependent on RNF8 and RNF168. The recombination substrates are diagrammed on the right with details described previously [33], [35], [37]. **A**. 293/HW1 cells transfected with indicated siRNAs (bottom grid) followed by transfection of the I-SceI expression plasmid to induce DSB. After 3 days, the repair efficiency was measured by applying quantitative real-time PCR on extracted DNA, represented by the percentage on the *Y* axis. In each experiment, the yield of conservatively repaired DNA was normalized relative to the result from the control siRNA transfection. Results (+/− SEM) are from three independent experiments. NT indicates no transfection of the I-SceI expressing plasmid. **B**. same as in panel A except that siRNA targeting the *53BP1* 3′UTR was transfected in combination with the wild-type 53BP1 expression plasmid or an empty vector, as indicated. **C–E**. cells were subjected to two rounds of transfections as in A and the percentages of GFP-positive cells were determined by flow cytometry. In each experiment, the percentage of GFP-positive cells from control siRNA transfections was set equal to 1, and the fraction of GFP-positive cells was determined relative to the control siRNA to measure Alt-NHEJ, HDR, and SSA, respectively. **F**. immunoblots show the depletion of indicated protein by RNAi interference, or the expression of 53BP1 protein by plasmid transfection. H4, RHA, β-actin or α-tubulin were loading controls.

53BP1 regulates DNA double-strand break (DSB) repair in the NHEJ pathway, but its specific function is unclear. To explore the role of 53BP1 in a specific pathway of DSB, we used cell lines that contain integrated into their genomes recombination substrates that specifically probe the conservative-NHEJ (C-NHEJ), alternative-NHEJ (Alt-NHEJ), homology directed repair (HDR), and single-strand annealing (SSA) repair pathways [33]–[35], [37] (diagrammed in Figure 1A and C–E, *right*). The general strategy is to deplete by siRNA transfection 53BP1 or another factor, followed by transfection of a plasmid that expresses the rare-cutting restriction endonuclease I-SceI, which simulates a DSB at a specific site. 293/HW1 cells [37] contain a DNA substrate with two neighboring I-SceI sites in the genome for which repair by the C-NHEJ pathway can be measured by the precise joining of the DNA ends following I-SceI expression (Figure 1A, *right*). The concentration of DNAs repaired by C-NHEJ was measured by real-time PCR using an oligonucleotide probe that spans the break site. Depletion of ligase IV, which is known to affect the NHEJ repair frequency, reduced the C-NHEJ repair to 18% relative to the control siRNA. To test 53BP1 in the C-NHEJ pathway, 53BP1 was depleted in 293/HW1 cells, and we found that repair efficiency decreased to approximately 41% relative to the control siRNA (Figure 1A). Transfection of another siRNA targeting the *TP53BP1* 3′ untranslated region (3′UTR) sequence to deplete endogenous mRNA resulted in a decrease of C-NHEJ to 70% relative to the control. This siRNA reproducibly yielded less inhibition of the C-NHEJ than did the siRNA targeting the 53BP1 coding region. When this 3′-UTR specific siRNA was co-transfected with a plasmid expressing wild-type 53BP1 resistant to this siRNA, C-NHEJ repair efficiency was restored to 98% relative to the control, demonstrating the specificity of the siRNA depletions (Figure 1B). This observation of the role of 53BP1 in C-NHEJ is consistent with observations that 53BP1 promotes fusions of deprotected telomeres via C-NHEJ repair of the telomeres during G1 phase [40].

Depletion of BRCA1, a protein that regulates multiple DSB repair pathways, reduced C-NHEJ repair efficiency to about 49%. Co-depletion of 53BP1 and BRCA1 depressed the ratio further to about 19%, indicating that in this repair pathway BRCA1 and 53BP1 were not antagonistic, but rather each functioned to independently stimulate C-NHEJ.

The E3 ubiquitin ligases RNF8 and RNF168 have been demonstrated to be required for 53BP1 and BRCA1 localization at DSBs in an ubiquitination-dependent manner [17]. We depleted RNF8 or RNF168 by siRNA in 293/HW1 cells, resulting in a decrease in the C-NHEJ to 14% and 31%, respectively (lanes 6, 7). Co-depletion of both RNF8 and RNF168 did not have any additive effect compared to either single depletion (lane 10), indicating the epistatic role of RNF8 with RNF168 in the C-NHEJ pathway. We then tested whether RNF8 or RNF168 also regulates 53BP1 function in the C-NHEJ process. Co-depletion of RNF8 or RNF168 with 53BP1 had no additive effect relative to single depletion, consistent with the concept that RNF8 and RNF168 function in the same NHEJ pathway as 53BP1. These results along with prior observations [19], [20], [22] indicate that RNF8 and RNF168 are epistatic with 53BP1, which functions in C-NHEJ in a RNF8/RNF168-dependent manner.

We next tested 53BP1 function in the Alt-NHEJ pathway using a cell line, HeLa-EJ2, which has integrated in its genome a recombination substrate that is repaired by Alt-NHEJ to generate a functional GFP gene [33] (Figure 1C, *right*). BRCA1 depletion caused a decrease to 55% relative to the control siRNA, consistent with the literature [41]. Depletion of 53BP1, RNF8, or RNF168 each had minimal effect on Alt-NHEJ, which was not statistically significant (Figure 1C). We had anticipated that depletion of 53BP1 would enhance Alt-NHEJ function since the Alt-NHEJ pathway depends on resection of DNA ends, an activity thought to be inhibited by 53PB1. We suggest that the major product of this assay depends on resection of a short stretch of DNA, ∼35 bp, and was not inhibited by 53BP1. Co-depletion of 53BP1 with RNF8 or RNF168 had little impact on Alt-NHEJ though co-depletion of both RNF8 and RNF168 did impair the Alt-NHEJ repair efficiency compared to control (lanes 8–10). This last result suggests that RNF8 and RNF168 have redundant function in the Alt-NHEJ pathway, but it is independent of 53BP1.

We compared 53BP1 to BRCA1 function in homologous recombination, which has a major pathway of HDR and the minor SSA pathway. We utilized HeLa-DR cells and HeLa-SA cells to conduct the HDR and SSA assays, respectively. Repair by each pathway is measured by the conversion of cells to GFP-positive (Figure 1D and E, *right*). Depletion of 53BP1 had no effect in HDR (Figure 1D) but increased SSA (Figure 1E). The increase in SSA activity in 53BP1-depleted cells probably reflected relief from 53BP1-mediated inhibition of DNA resection needed for the SSA pathway. BRCA1 depletion affected both homologous recombination pathways (Figure 1D, E). Co-depletion of 53BP1 partially rescued the deficit caused by single depletion of BRCA1 in HDR or SSA, and this is consistent with the literature [42]. RNF8 and RNF168 depletions resulted in a statistically significant decrease in HDR but not in SSA. Co-depletion of both RNF8 and RNF168 had an additive effect in HDR and caused a decrease of repair efficiency. Co-depletion of 53BP1 and either RNF8 or RNF168 did not show any additive effect in both assays. BRCA2, which suppresses SSA [35], [43], was used as a negative control (Figure 1E). Depletions of protein by siRNAs were confirmed by immunoblot (Figure 1F).

In summary for Figure 1, we investigated the role of 53BP1 in DNA DSB repair pathways and identified that it functions positively in C-NHEJ pathway, has no function in either HDR or Alt-NHEJ, and 53BP1 suppresses SSA. 53BP1 functions in the same C-NHEJ pathway as RNF8 and RNF168, which are known to regulate 53BP1 localization to sites of DSBs. Combined with prior studies, these results suggest that 53BP1 positively regulates C-NHEJ pathway in a RNF8- and RNF168-dependent manner.

### 53BP1 is destabilized upon irradiation damage

**Figure 2:**
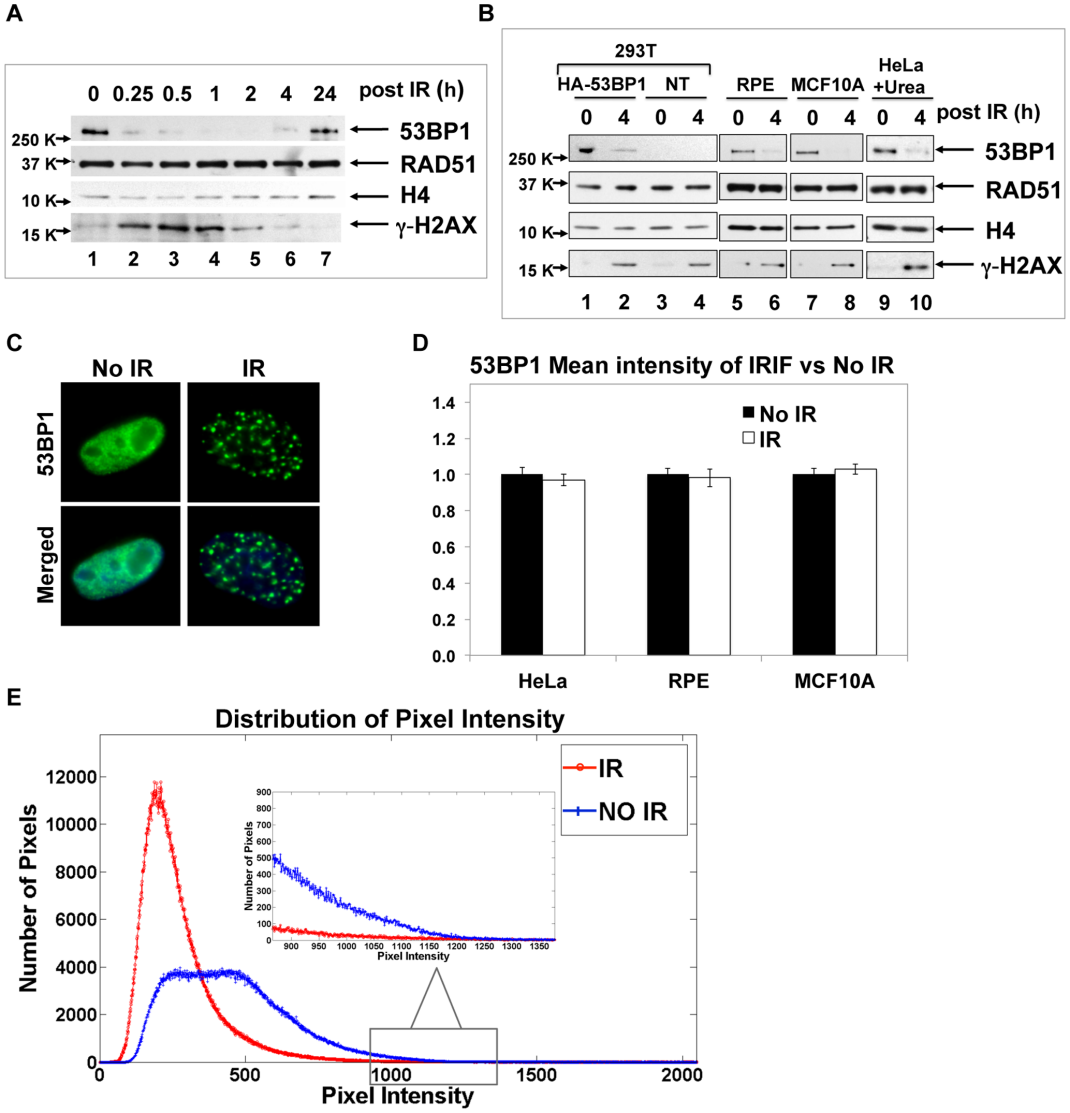
53BP1 protein abundance decreases upon irradiation. **A**. HeLa cells were subjected to 10 Gy X-rays and total cell lysates were prepared in 2% SDS containing buffer at the indicated time points. Immunoblots of indicated protein were shown. H4 was a loading control. The positions of the molecular mass markers in kDa (K) are indicated at the *left*. **B**. HA-53BP1 plasmid was transfected into 293T cells, and 48 h post-transfection, cells were irradiated and extracted in SDS containing buffer; NT indicates no transfection (lanes 1–4). Retinal pigment epithelia cells (RPE; lanes 5, 6) and normal mammary epithelial cells (MCF10A; lanes 7, 8) were exposed to 10 Gy X-rays and total cell lysates were prepared in SDS containing buffer 4 h after irradiation. HeLa cells were exposed to 10 Gy irradiation similarly and total cell lysates were extracted 4 h post-IR in buffer containing 2% SDS and 8 M urea (lanes 9, 10). Immunoblots were developed using anti-HA antibody to detect HA-tagged 53BP1 protein in 293T cells (lane 1–4), and antibody specific for 53BP1 to recognize endogenous 53BP1 in RPE, MCF10A and HeLa cells (lane 5–10). **C**. HeLa cells were subjected to immunofluorescence microscopy 4 h post-irradiation (10 Gy). Cells were stained for 53BP1 (green; *top*) and merged with DAPI stain of DNA (blue; *bottom*). **D**. Similar to the immunofluorescence microscopy experiment in panel C, RPE and MCF10A cells were stained for 53BP1 protein before and after irradiation. For the immunofluorescence images, 100 nuclei were analyzed in HeLa cells, and 30 nuclei were analyzed in RPE or MCF10A cells using ImageJ software. Mean intensity of 53BP1 signal at foci regions in each nucleus in irradiation sample were scored and normalized according to the mean intensity of diffuse 53BP1 signal in no irradiation sample. Results (mean +/− SEM) were shown for three cell lines. **E**. The distribution of pixel intensities of 53BP1 nuclear stain in HeLa cells, as in panel C, was plotted for No IR (blue) and IR (red). Results were shown from 80–100 nuclei in each sample. Pixel intensities within the range of 850–1400 were shown in the graph inset.

53BP1 forms ionizing radiation induced foci (IRIF) in response to DNA damage, and the protein has highest abundance during G1 phase, a stage in the cell cycle associated with NHEJ activity [44]. To investigate 53BP1 protein dynamics in response to irradiation-induced DNA damage, we evaluated changes in 53BP1 protein bulk level four hours post-irradiation (10 Gy). Surprisingly, the 53BP1 protein level decreased markedly compared to the non-irradiated control (Figure 2A, lanes 1–6). In this experiment we were careful to extract all of the 53BP1 protein in the HeLa cells by including 2% SDS in the lysis solution followed by a thorough sonication and heating at 100°C. Thus, the absence of 53BP1 protein in the irradiated cell lysates was not due to compartmentalization of the protein into an insoluble fraction. In contrast to 53BP1, the protein abundance of the homologous recombination factor RAD51 did not change upon irradiation. The DSB damage signal sensor γ-H2AX was used as a positive control and histone H4 served as the loading control. We found that 53BP1 protein levels decreased to very low concentration as early as 15 minutes following ionizing radiation and were restored after 24 hours (Figure 2A, lanes 2–7). In order to rule out that the detection of degradation of 53BP1 protein was specific for the antibody, we also tested for degradation when using HA-tagged 53BP1 protein expressed from a transiently transfected plasmid. Upon irradiation of 293T cells transfected with a HA-53BP1 expression plasmid and lysis in SDS containing buffer, detection of the HA-53BP1 via its epitope tag also revealed a substantial decrease in protein levels 4 h post-irradiation (Figure 2B, lanes 1, 2). Detection of the HA-53BP1 protein by the anti-HA antibody was specific for transfected samples (Figure 2B, lane 3, 4). Furthermore, in two non-cancer cell lines, human retinal pigment epithelium (RPE) cells and normal mammary epithelium MCF10A cells, the endogenous 53BP1 protein levels in 2% SDS lysates were diminished 4 h post-IR (Figure 2B, lane 6, 8), consistent with the results from the HeLa and 293T cell lines. To rule out any potential artifact due to the incomplete extraction of 53BP1 protein from chromatin, urea containing buffer (8 M urea and 2% SDS in phosphate-buffered saline) was used as a reliable means for the complete extraction of the chromatin-bound protein followed by a thorough sonication and heating at 100°C. 53BP1 protein extracted by this method showed a greatly diminished level 4 h post-IR in HeLa cells, compared to no irradiation (Figure 2B, lane 9, 10), further confirming the above results. The level of repair protein RAD51 did not change upon irradiation. γ-H2AX was a positive damage sensor and histone H4 was used as a loading control in 293T, RPE, MCF10A and HeLa cell lysates (Figure 2B).

Since 53BP1 is a DSB repair protein, we were surprised to observe that its protein abundance sharply decreased as early as 15 minutes post DNA damage. It is well known that 53BP1 protein forms prominent IRIF after ionizing radiation, and this seemed to contradict the results from post-irradiation whole cell lysates. To confirm the immunoblot results, we evaluated 53BP1 IRIF 4 h post-IR in HeLa cells and then utilized image analysis to measure the mean intensity of 53BP1 signal at IRIF regions in the nuclei. In non-irradiated cells, 53BP1 protein was diffusely abundant in nuclei, whereas upon ionizing radiation, 53BP1 was everywhere diminished except at DNA damage sites (Figure 2C). Staining 53BP1 IRIF in RPE cells or MCF10A cells, showed the same observation in comparison with non-irradiated cells (Figure S1 in File S1). We quantified the relative mean fluorescence intensity of the 53BP1 signal at foci regions from 100 irradiated nuclei in HeLa cells and 30 irradiated nuclei in human RPE cells or MCF10A cells by using ImageJ software. Similarly, we measured the 53BP1 mean intensity throughout the non-irradiated nuclei as 53BP1 diffusely stains the nuclei. The comparison between irradiated and non-irradiated nuclei showed no difference in the mean intensity of 53BP1 signal nucleoplasm before IR and at IRIF regions in the three cell lines (Figure 2D). This result together with observation in Figure 2C and Figure S1 in File S1 suggests that 53BP1 foci appearance upon IR is due in large part to the degradation of diffuse 53BP1 in the nucleoplasm at undamaged regions.

Using the above image analysis method, we have assessed 53BP1 signal at damage sites represented by the mean intensity of fluorescence signal at IRIF, which is computed by the total intensity of pixels based on an area unit. There is a potential for bias in this approach in defining the regions for measuring the mean pixel intensity. We thus developed an unbiased method to assess the intensities of 53BP1 signal at all individual pixels within the nuclei (Figure 2E). 80–100 nuclei were outlined by DAPI stain, and the DAPI stained areas were analyzed for 53BP1 signal in irradiated or non-irradiated samples in HeLa cells using an algorithm in MATLAB R2013a. We plotted the distribution of all individual pixel intensities in the nuclei, and observed that for the irradiated nuclei, most of the pixels were in the very low intensity group (red trace in Figure 2E) as would be expected for foci, whereas the 53BP1 stain in the non-irradiated nuclei was more evenly distributed for pixel intensity and with fewer low intensity pixels but more high intensity pixels (blue trace in Figure 2E). Even for the foci in the irradiated sample with only a few pixels with very high intensity (representing bright dots of small area), the diffuse 53BP1 stain in non-irradiated cells was as intense (Figure 2E, inset). If the appearance of 53BP1 IRIF resulted from the movement of protein from the nucleoplasm to damage sites, then we would have anticipated that post IR a small number of pixels would gain intensity of 53BP1 stain above the baseline. Such an expectation was the opposite of what was observed: following IR most pixels had a decrease in 53BP1 staining intensity and foci did not have higher intensity stain when compared to the unirradiated nucleus. This result was consistent with data shown in Figure 2D and clearly suggests that the appearance of 53BP1 foci was due in large part to its degradation in the nucleoplasm. We also measured γ-H2AX signal by image analysis in both conditions (No IR versus IR, data not shown) and the protein level increased upon irradiation, consistent with observations by immunoblotting (Figure 2A). Though the appearance of 53BP1 at IRIF suggests recruitment from the nucleoplasm to DNA damage sites, these results presented here suggested instead that 53BP1 binding to damage sites renders it resistant to the universal degradation after ionizing radiation.

**Figure 3:**
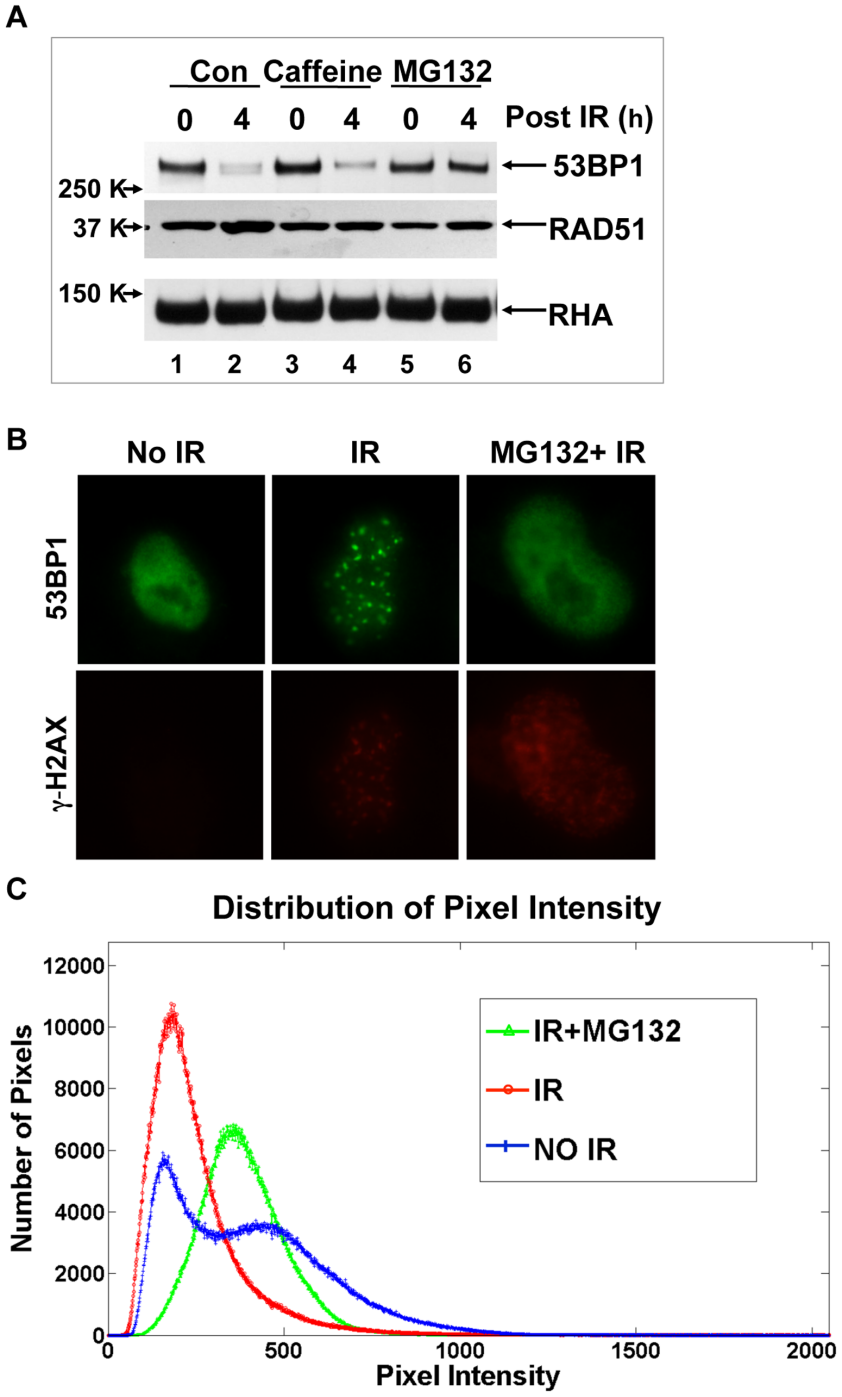
53BP1 protein content decreases upon irradiation is due to protein degradation. **A**. Before subjection to 10 Gy X-rays, HeLa cells were treated with medium alone (Con; lanes 1, 2), caffeine (10 mM; lane 3, 4) or with MG132 (20 µM; lane 5, 6). 4 h post irradiation, cell lysates were prepared for immunoblots. **B**. HeLa cells were treated with or without MG132 as in panel A, and immunofluorescence microscopy was applied after 4 h irradiation (10 Gy) as in Figure 2C. **C**. Image analysis of cells as shown in panel B was done as in Figure 2E. The distribution of pixel intensity was plotted for each sample.

Next we tested if the decrease in 53BP1 protein abundance in response to irradiation in HeLa cells was due to proteasome-dependent degradation. Inclusion in medium of MG132, the proteasome inhibitor, blocked the decrease in 53BP1 protein concentration upon irradiation whereas inclusion of caffeine in the medium, an ATM inhibitor, did not. This result indicated that protein degradation, but not loss of ATM-dependent phosphorylation of 53BP1, caused the decrease of 53BP1 level after irradiation (Figure 3A). 53BP1 immunofluorescence following irradiation in HeLa cells was then analyzed in the presence of MG132, which resulted in the same diffuse 53BP1 pattern as observed in the absence of irradiation (Figure 3B). Image analysis confirmed that the IRIF do not have higher intensity 53BP1 stain than did the unirradiated sample or the IR plus MG132 sample (Figure 3C), indicating that 53BP1 protein was degraded upon irradiation.

### Depletion of RNF8 or RNF168 blocks 53BP1 degradation upon irradiation

**Figure 4:**
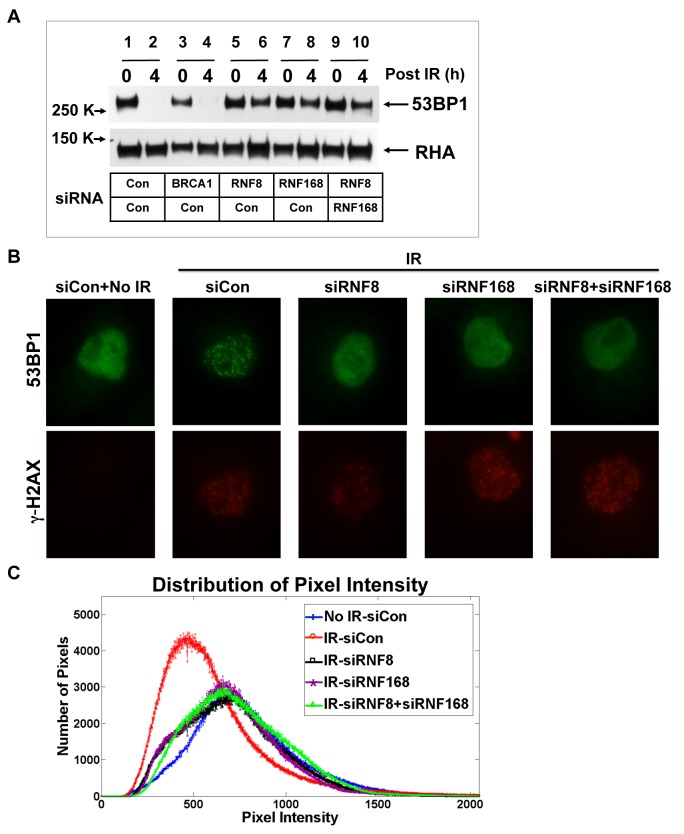
53BP1 degradation upon irradiation is regulated by RNF8 and RNF168. **A**. HeLa cells transfected with two different siRNAs (indicated in the grid) were treated with 10 Gy X-rays. 4 hours post-IR cell lysates were prepared for 53BP1 immunoblot analysis. RHA served as a loading control. **B**. Immunofluorescence microscopy analysis of cells from panel A were stained for 53BP1 (green) and γ-H2AX (red). **C**. distribution of pixel intensity was analyzed from microscopic images in panel B.

RNF8 and RNF168 are E3 ubiquitin ligases that mediate the conjugation of ubiquitin multimers on histone H2A via the degradation-independent lysine-63 side-chain of ubiquitin and via this activity recruit other proteins, such as 53BP1 and BRCA1, to the sites of DNA damage [17], [19]–[22], [26]. We tested the possibility that these two enzymes are involved in 53BP1 protein degradation. Indeed, depletion of RNF8 or of RNF168 from HeLa cells and following irradiation-induced DNA damage, 53BP1 degradation was blocked (Figure 4A, lanes 6, 8, 10). Depletion of BRCA1, another E3 ubiquitin ligase involved in the DNA damage response, did not affect the 53BP1 protein level, indicating RNF8/RNF168 had a specific role in the control of 53BP1 protein levels upon irradiation (Figure 4A, lane 4). Consistent with the model that 53BP1 is degraded dependent on RNF8 and RNF168, the 53BP1 protein remained diffusely localized in the nucleus in HeLa cells in which these factors were depleted (Figure 4B). The distribution of pixel intensity in immunofluorescence images showed that depletion of RNF8 and/or RNF168 had a similar distribution pattern as no irradiation, though with more intense pixels (Figure 4C), suggesting that RNF8 and RNF168 regulate proteasome-dependent degradation and IRIF formation of 53BP1 in response to irradiation-induced DNA damage.

### 53BP1 turnover is accelerated upon irradiation damage

**Figure 5:**
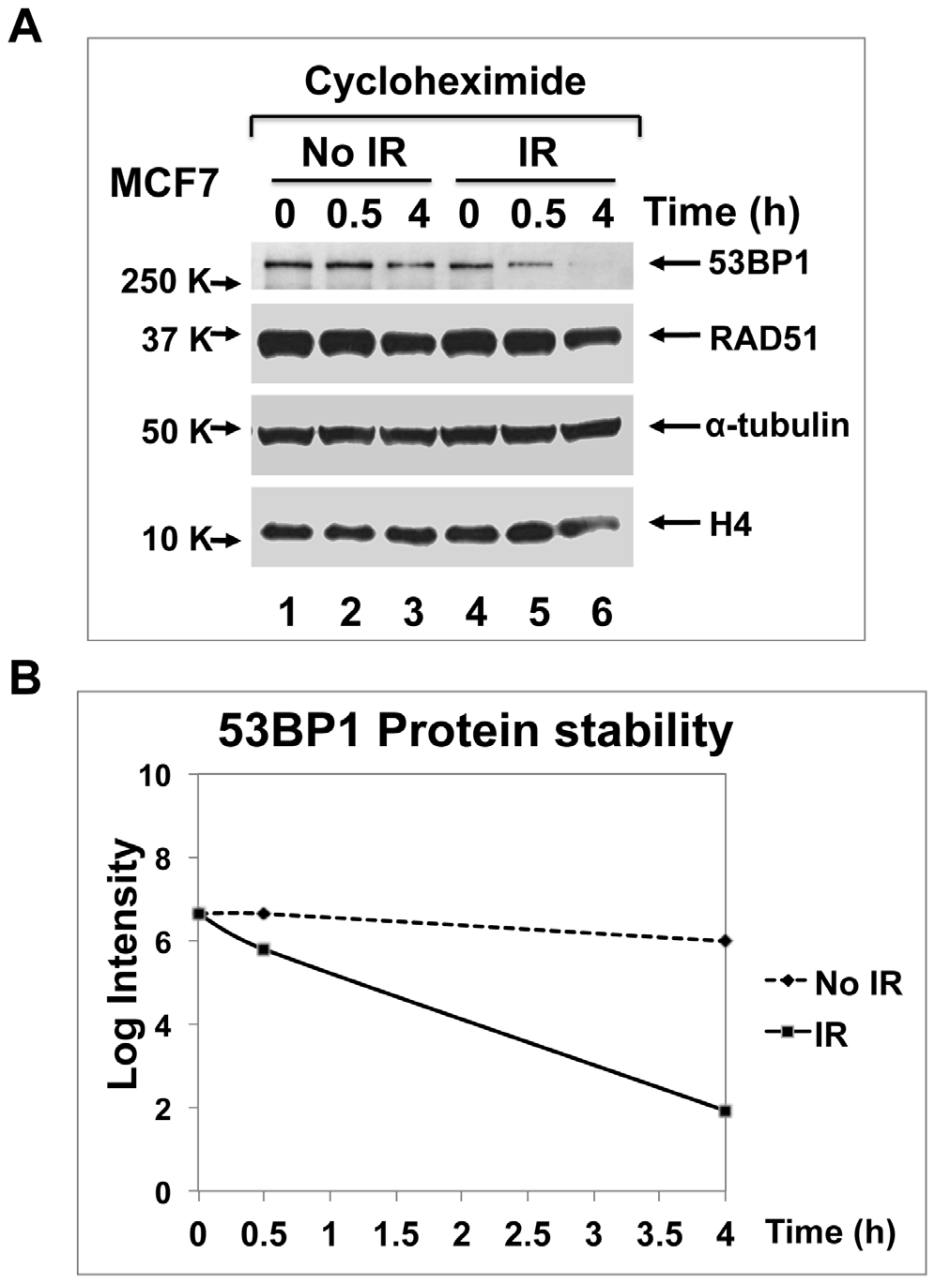
53BP1 turnover is accelerated upon irradiation. **A**. Cycloheximide (100 µg/mL) was added to MCF7 cells with or without irradiation (10 Gy) and total cell lysates were prepared in SDS containing buffer according to the indicated time course (0, 0.5 and 4 h) and analyzed by immunoblotting as indicated. **B**. 53BP1 protein signal in immunoblot in A was measured by densitometry in each sample.

Since 53BP1 protein abundance changed following irradiation, we speculated that irradiation shortened the 53BP1 protein half-life. Using MCF7 (Figure 5) or HeLa cells (Figure S2 in File S1), we blocked new protein synthesis by the addition of cycloheximide to tissue culture media and made whole cell lysates in 2% SDS containing buffer. In the absence of IR, protein levels were stable (Figure 5A, lanes 1–3). By contrast, following IR, 53BP1 turnover was apparent as early as 30 min post-IR, and the protein level decreased to 4% 4 hours post IR (Figure 5A, B). By contrast, RAD51 half-life was not affected by IR (Figure 5A). The results together implicated that ionizing radiation accelerates 53BP1 protein turnover via the proteasome-dependent pathway.

### 53BP1 is protected from degradation at damage sites

**Figure 6:**
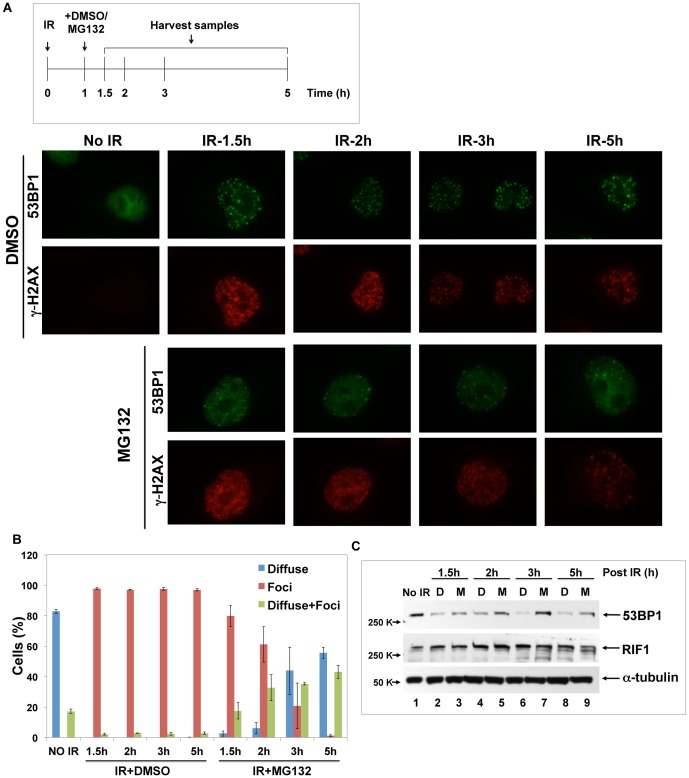
53BP1 is degraded except when bound to a damage site. **A**. *upper* panel shows the workflow of the experiment. 1 h after 10 Gy X-ray irradiation was applied to the HeLa cells, DMSO or MG132 (20 µM) was added to the media. At time points 1.5 h, 2 h, 3 h, and 5 h post-IR, cells were either fixed for microscopy (A, *bottom*) or lysed for immunoblot analysis (C). **B**. in each sample, the percentage of the cells that have diffuse 53BP1 stain (blue), 53BP1 foci (orange) or diffuse 53BP1 stain with foci (green) was quantified (mean ±SEM; N = 3). **C**. cell lysates taken from panel A were subjected to immunoblot for 53BP1 and RIF1 stain. Samples were treated with DMSO vehicle (D, even lanes) or MG132 (M, lanes 3, 5, 7, 9). Tubulin was a loading control.

Previous reports had suggested that 53BP1 is recruited to damage sites in an RNF8/RNF168 dependent manner [19], [22]. We speculated that after 53BP1 stably localizes and binds to DSBs at chromatin at time points as early as four minutes post-irradiation [22], degradation of 53BP1 rather than the protein movement to the sites of DSBs, leads to prominent 53BP1 focus formation. To differentiate between movement of the protein versus degradation of bulk 53BP1, we irradiated HeLa cells and after foci formed we blocked proteasome-mediated degradation. If 53BP1 protein relocated within the nucleus, then foci would remain even if the proteasome were blocked. If, on the other hand, the 53BP1 bound to the DNA damage site was stabilized then the foci would be surrounded by diffuse 53BP1 as new protein was synthesized. MG132 was added to the cells 1 hour post-irradiation and a series of times points were analyzed to observe the 53BP1 foci at the damage sites. (Refer to time-line in Figure 6A, *top*.) In the absence of the MG132, 53BP1 containing IRIF were apparent at all post-IR time-points analyzed. By comparison, in the cells in which MG132 was added to the medium one hour post-IR, foci were still apparent, but these IRIF were in the presence of diffuse 53BP1 stain at late time points (Figure 6A, *bottom*). As an indication of the diffuse 53BP1 localization, in the presence of MG132 the nucleoli become apparent as holes in the diffuse pattern. The results from this experiment were quantified in Figure 6B and show that MG132 treated cells primarily had diffuse nuclear 53BP1 stain or diffuse stain with foci, suggesting that degradation event in the nucleoplasm happened prior to MG132 treatment, and the diffuse stain was due to the appearance of newly synthesized 53BP1. When MG132 was added to the cells prior to IR, no foci were apparent (Figure 3B). These results are most consistent with a model in which 53BP1 is continuously synthesized at a high rate, and post-IR it is rapidly degraded via the ubiquitin-proteasome system (Figure 5A, B). Only at sites of DNA damage is it protected from ubiquitin-dependent degradation. Immunoblot results of 53BP1 protein from experiment in Figure 6A, *top* were consistent with the notion that 53BP1 accumulated to high levels when in the presence of MG132 (Figure 6C). By comparison, the concentration of the downstream effector RIF1 did not change following DNA damage (Figure 6C). Together, these data suggest that following ionizing radiation, 53BP1 is rapidly synthesized and rapidly degraded except when bound to repair sites.

### 53BP1 stability is important for RIF1 recruitment

**Figure 7:**
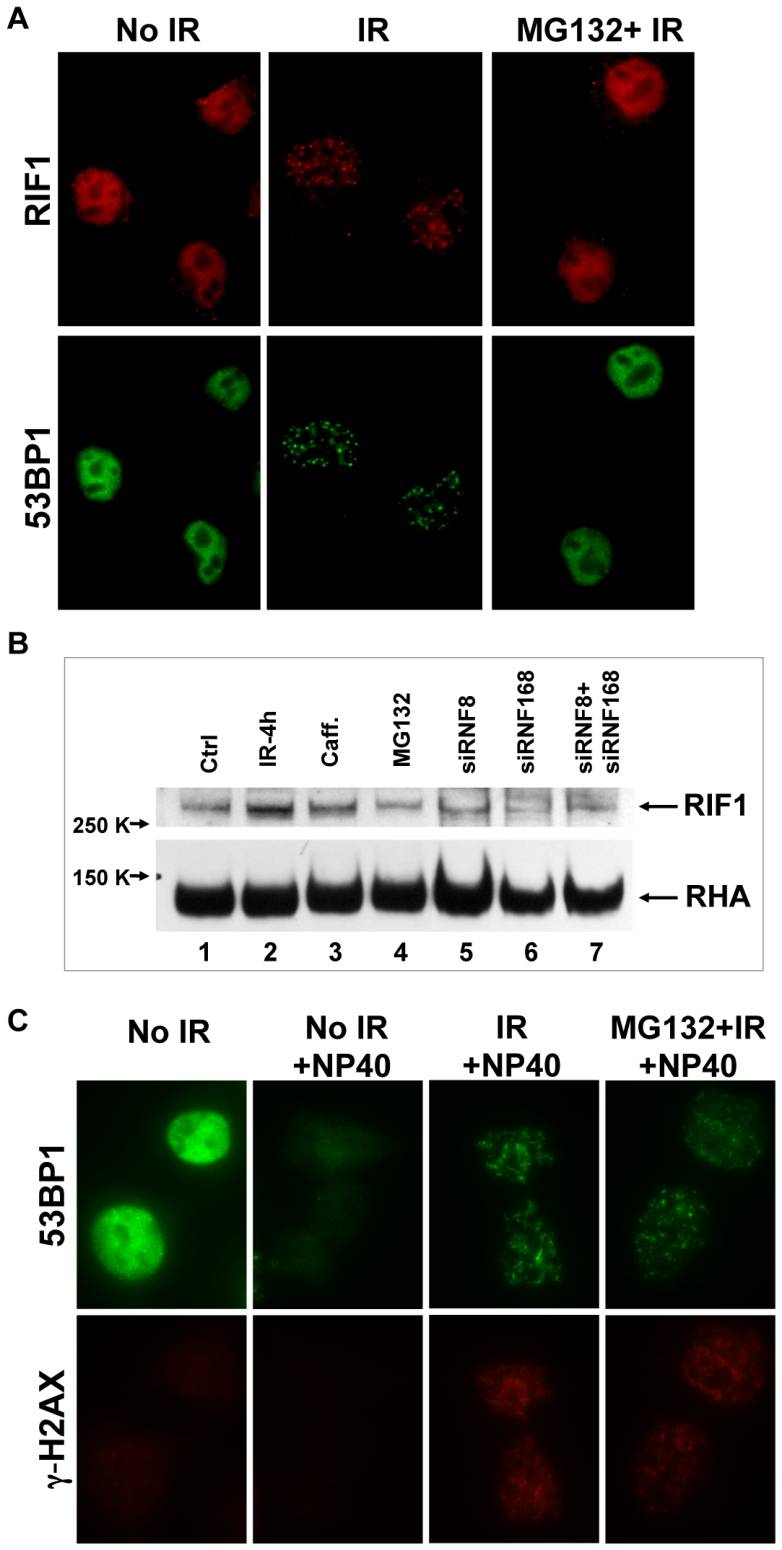
Inhibition of 53BP1 degradation causes failure to recruit RIF1 to the DSB sites. **A**. HeLa cells were treated with or without MG132 (20 µM) before exposure to 10 Gy X- irradiation. 4 h post-IR, cells were fixed for immunofluorescence microscopy as indicated. **B**. different treatments were applied to HeLa cells and immunoblot for RIF1 was done. Ctrl, no treatment (lane 1) or4 h post 10 Gy-IR (lanes 2–7). Additional treatments included caffeine (10 mM; lane 3) MG132 (20 µM; lane 4), siRNA specific for RNF8 (lane 5), siRNA specific for RNF168 (lane 6), and mixed siRNA specific for both RNF8 and RNF168 (lane 7). **C**. MG132 (20 µM) was included in medium 30 minutes prior to exposure to 10 Gy X-ray irradiation. At 4 h post-IR, HeLa cells were extracted *in situ* with cell extraction buffer (Material and Methods) on ice for 15 min (+NP40) or not extracted. Cells were fixed and stained for immunofluorescence microscopy as above.

RIF1 is the only known DNA damage repair factor that requires 53BP1 for its recruitment to damage sites [45], and which indirectly depends on RNF8 and RNF168 [46]. We tested if inhibition of 53BP1 protein degradation affects RIF1 association with IRIF. In contrast to 53BP1, irradiation did not affect RIF1 protein levels detected from immunoblots of lysates from HeLa cells (Figure 7B, lane 2). Inclusion of MG132 abolished RIF1 association with IRIF in HeLa cells (Figure 7A) without changing its protein level (Figure 7B, lane 4). Similarly, caffeine or RNF8 and/or RNF168 depletion did not affect RIF1 abundance (Figure 7B, lane 3, 5–7 and [45]).

To test whether the inhibition of RIF1 IRIF in the presence of MG132 was directly associated with the failure to degrade the unbound bulk 53BP1, but not due to the impaired ubiquitin-dependent DSB signaling, we modified the immunofluorescence staining protocol to include a detergent extraction step prior to fixation. Following IR in the presence of MG132 in HeLa cells, the unbound bulk 53BP1 protein within the nucleus was removed by using cell extraction buffer containing 0.5% NP40 and 300 mM NaCl, and then cells were fixed and stained as usual. We observed chromatin-bound foci of 53BP1 that co-localized with **γ**-H2AX stain after irradiation and in the presence of MG132 (Figure 7C). The NP40 in the presence of 300 mM NaCl was sufficient to remove the loosely tethered bulk 53BP1 and reveal 53BP1 bound to the damage sites, consistent with a previous observation [47]. This result indicated that proteasome inhibition 30 minutes prior to DNA damage did not abrogate tight binding of 53BP1 to damage sites in chromatin. While prior studies had suggested that the RNF8-mediated degradation of KDM4A/JMJD2A was required to expose H4K20me2 for the recruitment of 53BP1 to DNA damage sites [16], [23], [24], our result revealed that the block due to KDM4A/JMJD2A binding to the chromatin was not absolute, and 53BP1 could still bind to the damage sites. We conclude from Figure 7C that proteasome inhibition by MG132 does not abolish 53BP1 association with IRIF, but blocks the degradation of the unbound bulk 53BP1, accounting for the failure to recruit RIF1 to the damage sites.

Taken together with data of RNF8/RNF168 regulation on 53BP1 function, stability and IRIF, we conclude that 53BP1 is initially recruited to damage sites by RNF8 and RNF168. The same regulators then degrade 53BP1 and thereby ensure proper 53BP1 protein concentration within the nucleus to recruit the downstream response factor RIF1 to damage sites for further efficient repair, consistent with the results that 53BP1 functions in DSB repair (the C-NHEJ pathway) dependent on RNF8 and RNF168 (Figure 1A). If 53BP1 degradation fails to occur and the protein remains in high concentration throughout the nucleus, then RIF1 fails to bind to the DNA lesion. These results are consistent with there existing in the nucleus of a cell two pools of 53BP1, a small pool of 53BP1 bound to chromatin at the site of DNA damage, and a large pool of unbound or bulk 53BP1 in the nucleoplasm. Unbound 53BP1 is, in essence, a decoy that inhibits the signal from 53BP1 bound to the damage site.

## Discussion

53BP1 is a DSB repair protein previously identified to influence the NHEJ process [14], [16], though its specific function had not been clearly defined in this pathway. In this study, we found: 1) 53BP1 positively regulates the C-NHEJ pathway in a RNF8- and RNF168-dependent manner; 2) 53BP1 has no effect on the end resection dependent pathways of Alt-NHEJ pathway or on HDR, but it did suppress the end resection dependent SSA pathway; 3) the localization of 53BP1 at sites of DSBs is accompanied by the ensuing removal of bulk 53BP1 from the nucleus except at sites of DNA damage; 4) RNF8 and RNF168 are each required for the proteasome-mediated degradation of bulk 53BP1 after DNA damage; and 5) failure to degrade bulk 53BP1 in the nucleoplasm results in the failure for RIF1 to localize appropriately to DNA damage sites.

### 53BP1 binding to DSB sites

The immunoblots of total 53BP1 from four different cell lines (HeLa, 293T, RPE and MCF10A) clearly indicate that most of 53BP1 is degraded following ionizing radiation (Figure 2A, B). This observation of regulation of 53BP1 protein stability must be linked with how it binds to DSBs. 53BP1 localization to the sites of DNA damage requires the recognition of histone methylation, in particular H4K20me2 [28] by its tandem Tudor domain [29], [30]. 53BP1 also binds to a second epitope, H2A ubiquitinated on Lys-15 (H2AK15ub), a product of RNF168 ubiquitination on chromatin, via its ubiquitin-dependent recruitment (UDR) motif. Initial recruitment of 53BP1 to DSBs also requires K63-linked ubiquitination of 53BP1 by RNF168 [32]. We suggest that bivalent binding of 53BP1 to epitopes on chromatin (H4K20me2 and H2AK15ub), or its K63-linked ubiquitination, may block the 53BP1 K48-linked ubiquitin targeted degradation, which ensues after 53BP1 localization to DSBs, thus distinguishing between bulk 53BP1 in the nucleoplasm and 53BP1 bound at the damage site. Since bulk 53BP1 is not bound to these chromatin epitopes, it would be susceptible to ubiquitination and degradation. Recruitment thus has a different mechanistic implication for the 53BP1 protein: rather than a movement of all of the 53BP1 in the nucleoplasm to the sites of DNA damage, following the early time points (4 min) when those molecules near the damage site move to the marked chromatin and stably bind [22], [48], most 53BP1 is then degraded. The stabilization signal at sites of damage (Figure 7C) is in effect a signal for appearance of prominent focus formation. Within the first 4 minutes following ionizing radiation, the RNF8 and RNF168 proteins create the binding sites, and 53BP1 binds to the sites on the chromatin [22]. We suggest that some limited movement of 53BP1 is taking place at these early time points so that the protein localizes to the DNA lesion and degradation of the bulk 53BP1 in the nucleoplasm would occur soon after localization. Plus, 53BP1 protein continues to be synthesized, allowing more protein to accumulate at the IRIF while some fraction of the protein continues to be degraded. This observation does not negate the prior observations that define the 53BP1 binding site on damaged chromatin, but rather amplifies our understanding of how the two mechanisms work in synergy to effect repairs.

53BP1 was shown to constitutively associate with chromatin in the absence of DNA damage independent of RNF8 [4], [49] and likely this portion of chromatin-bound 53BP1 might quickly move to the damage sites at the early time points as some molecules are in the neighborhood of a DSB.

Mapping the region of 53BP1 that is required for its degradation upon IR and the identification of the site(s) within 53BP1 at which K48-linked ubiquitination occurs will have great interest. Mutation of the K48-linked ubiquitin acceptor site on 53BP1 will enable the testing the impact of 53BP1 degradation on IRIF and DSB repair pathways.

Previous studies have shown that 53BP1 was transiently immobilized at the sites of damage by Fluorescence Recovery After Photobleaching (FRAP) experiments [38], [50]. It is possible that there is dynamic exchange between the IRIF-bound 53BP1 and newly synthesized protein consistent with prior observations [50].

Other prior studies have used laser micro-irradiation for generation of localized damage in cellular DNA, and 53BP1 localized to these DNA damage tracts [22], [38], [51]. We suggest that such a result does not contradict our current observations, but rather we posit that the damage induced by the laser micro-irradiation may have been local, but it induced a signal of the damaged state throughout the nucleus and causing bulk 53BP1 degradation. The implication for the mechanism of 53BP1 damage site binding is consistent with the observation that inhibition of the proteasome blocks 53BP1 degradation and blocks the appearance of 53BP1-containing IRIF in the nuclei (Figure 3B). When unbound bulk 53BP1 is removed from the nucleus by the detergent, tightly-bound 53BP1 reveals itself as IRIF in the presence of MG132, confirming that upon irradiation, 53BP1 is degraded everywhere with the exception of DNA damage sites where 53BP1 is bound and stabilized (Figure 7C). Excessive 53BP1 has been shown to be repressive to end-joining activity [16], and we suggest that this repressive activity may be due to the unbound pool of 53BP1 acting as a competitive inhibitor of the damage site bound 53BP1. Bulk 53BP1 prevents RIF1 from binding to the DNA in the undamaged state. After IR, bulk 53BP1 is degraded, and RIF1 is recruited to damage sites by bound 53BP1 to execute inhibition of end resection [15], [46], [52]. RIF1 is one of a few proteins identified to date that requires 53BP1 for its recruitment to DSBs [45] and is involved in C-NHEJ [46].

### 53BP1 versus BRCA1 function in DSB repair

53BP1 has been previously implicated as a competitor with BRCA1 in leading to opposite directions in the DSB repair process, NHEJ versus homologous recombination, respectively [13], [14], [46], [53]. Consistent with this notion, depletion of 53BP1 partially suppressed the effects of depletion of BRCA1 on homologous recombination by partially rescuing defects caused by the loss of BRCA1 (Figure 1D, E). 53BP1 suppressed the SSA pathway, but had no effect on HDR, however in each case its depletion could partially correct the defect due to depletion of BRCA1. On the other hand, depletion of BRCA1 caused deficits of varying magnitude to all four DSB pathways, including C-NHEJ, which was stimulated independently by both 53BP1 and by BRCA1, suggesting that the opposing function of BRCA1 versus 53BP1 is actually complex.

### The roles of RNF8 and RNF168

We found that 53BP1 specific function in the C-NHEJ process is RNF8- and RNF168-dependent since these factors regulated the degradation of 53BP1. This result is in agreement with a study that RIF1 accumulation at DSB sites is dependent on RNF8 and RNF168 [46]. RNF8 and RNF168 are the E3 ligases that conjugate ubiquitin to histones H2A and H2AX. These provide the binding site for 53BP1 at DSBs [29]. The simplest interpretation of the results would be that one of RNF8 or RNF168 has the additional activity of ubiquitinating bulk 53BP1 via the K48-linked side chain, marking it for degradation by the ubiquitin-proteasome system (UPS). Since depletion of either factor stabilizes 53BP1 as effectively as simultaneously depleting both, since RNF8 is upstream of RNF168, and since RNF168 interacts directly with 53BP1 [32], we suggest that RNF168 is the E3 ligase that directly targets 53BP1 for proteasome mediated degradation. Such a notion would imply that RNF168 can change its specificity for different E2 factors from one E2 that targets ubiquitin Lys-63 linkages when modifying H2A to another E2 that targets ubiquitin Lys-48 linkages when modifying bulk 53BP1. Alternatively, it is possible that an as yet undetermined E3 ubiquitin ligase is downstream of RNF8 and RNF168 and which mediates the K48-linked ubiquitination of the bulk 53BP1.

Our results indicate that RNF8 and RNF168 function differently in each DSB repair pathway. For the C-NHEJ pathway, RNF8, RNF168, and 53BP1 have an epistatic relationship since pairwise depletion in any combination of these three factors equally affected the repair rate as single depletion (Figure 1A). By contrast, simultaneous depletion of RNF8 and RNF168 had a more severe effect on the Alt-NHEJ pathway. We interpret this finding to mean that these two ubiquitin ligases function independently in this NHEJ pathway. Similarly, in the HDR pathway depletion of the RNF8 and RNF168 were additive in their effect. These results dissect out distinct interrelationships between these two ubiquitin ligases and with 53BP1, and these give clues to the mechanisms by which they act in the DSB repair.

In summary, this study found that following ionizing radiation, there are effectively two populations of 53BP1: stable DSB-bound 53BP1 and bulk 53BP1 protein that is degraded by the proteasome pathway. Degradation of 53BP1 prevents competition between bulk 53BP1 and the DSB-bound 53BP1 for the recruitment of RIF1 to DNA damage sites. In addition, it was found that 53BP1 functions positively in the sequence conserving precise NHEJ process dependent on RNF8 and RNF168, but 53BP1 has no role in the error prone Alt-NHEJ process. Loss of 53BP1 protein would thus be highly mutagenic since the error-prone NHEJ repair would predominate. This study also dissected how the RNF8 and RNF168 protein function in the four DSB repair pathways and indicate that relationships between these factors are pathway-specific.

## Supporting Information

File S1 **Supporting Figures. Figure S1. 53BP1 protein is everywhere diminished except at DNA damage sites.** RPE cells or MCF10A cells were subjected to immunofluorescence microscopy 4 h post-irradiation (10 Gy). Cells were stained for 53BP1 (green; *top*) and γ-H2AX (red; *bottom*). **Figure S2. In HeLa cells, 53BP1 turnover was accelerated upon irradiation.** Procedure in lane 3–8 was done as in Figure 5A except that HeLa cells were analyzed and two controls were included: no irradiation and irradiation (post 4 h IR) in lane 1 and 2.
(TIF)
