# Supplementary figures and images for: Regulation of 53BP1 Protein Stability by RNF8 and RNF168 Is Important for Efficient DNA Double-Strand Break Repair
Source: PLoS One. 2014 Oct 22;9(10):e110522. doi: 10.1371/journal.pone.0110522 (PMC4206297; doi:10.1371/journal.pone.0110522)

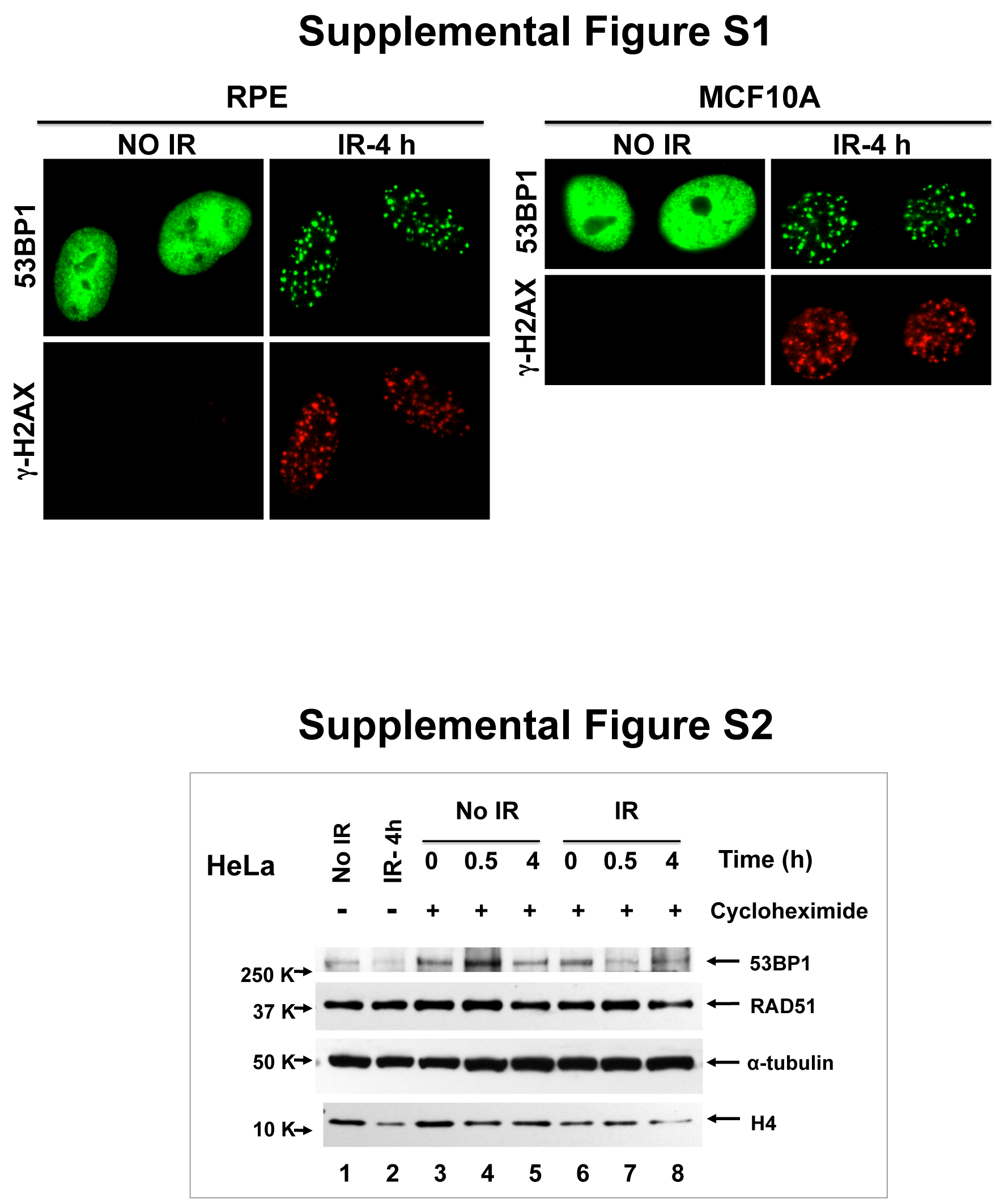

Supplement: File S1 — Supporting Figures. Figure S1. 53BP1 protein is everywhere diminished except at DNA damage sites. RPE cells or MCF10A cells were subjected to immunofluorescence microscopy 4 h post-irradiation (10 Gy). Cells were stained for 53BP1 (green; top) and γ-H2AX (red; bottom). Figure S2. In HeLa cells, 53BP1 turnover was accelerated upon irradiation. Procedure in lane 3–8 was done as in Figure 5A except that HeLa cells were analyzed and two controls were included: no irradiation and irradiation (post 4 h IR) in lane 1 and 2. (TIF) [file pone.0110522.s001.tif]
